# Supplementary material for: A New High-Throughput Screening Method to Detect Antimicrobial Volatiles from Metagenomic Clone Libraries
Source: Antibiotics (Basel). 2020 Oct 22;9(11):726. doi: 10.3390/antibiotics9110726 (PMC7690600; doi:10.3390/antibiotics9110726)
Supplement: Supplementary file 1 [file antibiotics-09-00726-s001.pdf]

antibiotics

MDPI

Supplementary material

A new high-throughput screening method to detect antimicrobial volatiles from metagenomic clone libraries

Franz Stocker [1,2+], Melanie M. Obermeier [1,2+], Katharina Resch [1,2], Gabriele Berg [1,2], Christina A. Müller Bogotá [1,2*]

[1] Institute of Environmental Biotechnology, Graz University of Technology, Austria; gabriele.berg@tugraz.at
[2] Austrian Centre of Industrial Biotechnology, Austria; info@acib.at
* Correspondence: christina.mueller@tugraz.at; Tel.: 0043 316 873 – 8310 (C.A.M.B.)
\+ These authors contribute equally to the paper

FIGURES

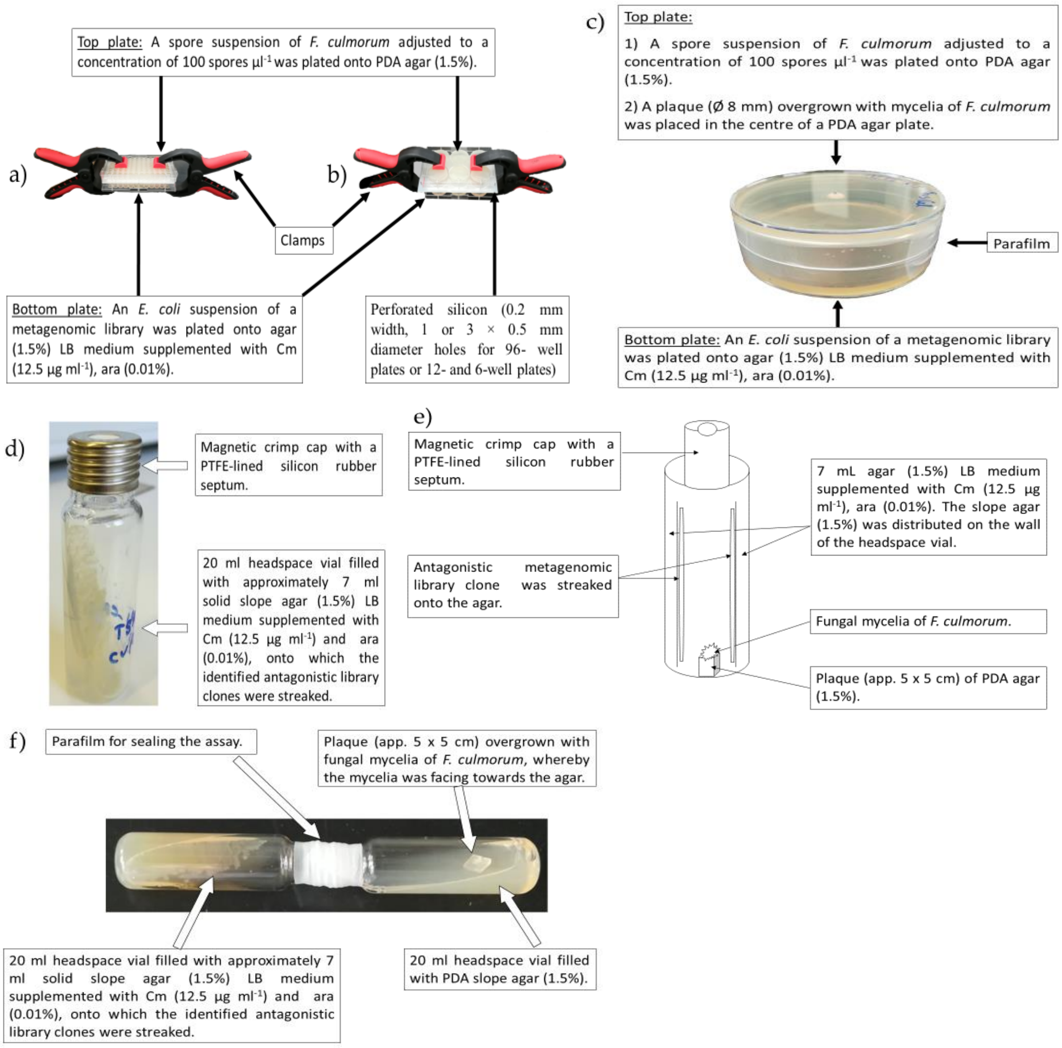

Figure S1: Illustration of the different assays. Set-up of the high-throughput Two Clamp VOCs Assay (htTCVA) (a), the Two Clamp VOCs Assay (TCVA) (b) and the Petri Dish VOCs Assay (PDVA) (c) for the high throughput screening and the set-up of single cultivation (d), one-vial co-cultivation (e) and separated co-cultivation (f) prior to the SPME GC-MS measurements.

De novo sequencing and gene annotation

Metagenomic DNA inserts of positive tested clones were sequenced by the Göttingen Genomics Laboratory (Göttingen, Germany) as previously described [1]. In short, using the Illumina MiSeq technology (Illumina, California, USA) 300 bp reads were generated, quality filtered with Trimmomatic v0.36 [2], assembled with SPAdes v3.10.0 [3] and genes annotated with Prokka [4].

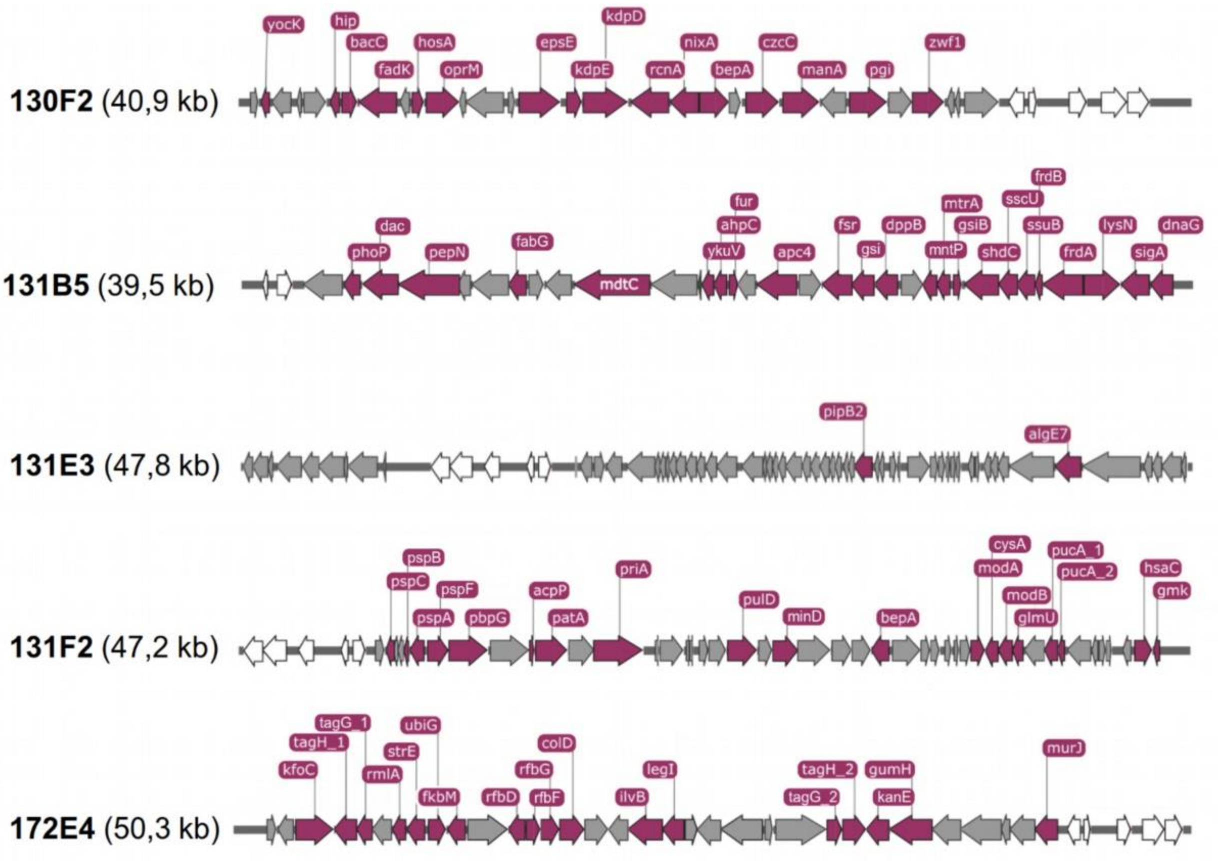

Figure S2: Insert maps of the fosmid clones. Sequences of fosmid clones displaying annotated genes (pink), unknown genes (grey) and genes from the fosmid backbone (white).

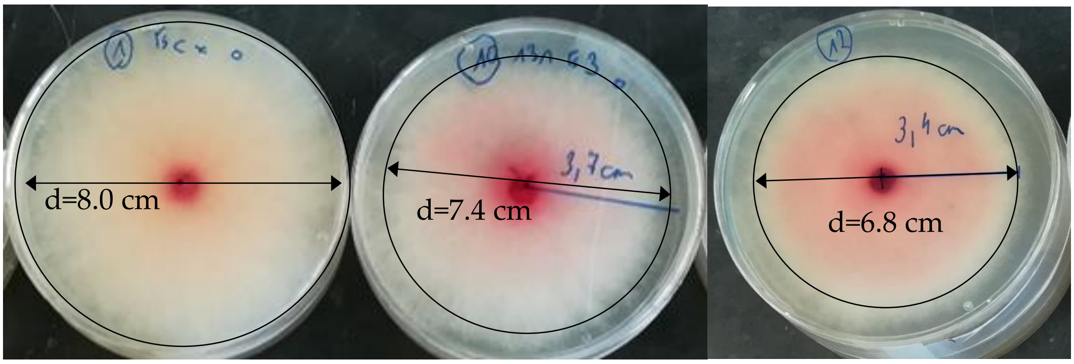

Figure S3. Volatile inhibition of *F. culmorum*. Growth inhibition of 7.5% by the metagenomic library clone *E. coli* EPI300 pCC2FOS 131 E3 (center and right) as compared to the empty vector control *E. coli* EPI300 pCC2FOS (left).

VOCs profiling through SPME GC-MS

(a)

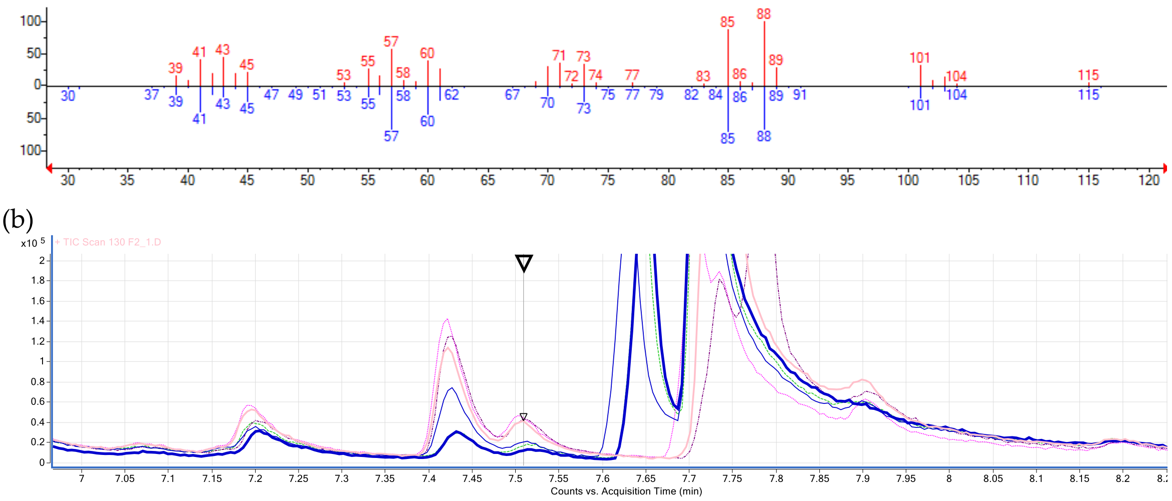

FIGURE S4. Detection of valeric acid. (a) Comparison of the recorded mass spectrum (red) and the spectrum of the NIST reference (blue) for valeric acid. (b) SPME GC-MS measurement displaying scans of the metagenomic clone (pink) as compared to the empty vector strain *E. coli* EPI300 pCC2FOS (blue and green) with sample size n=3. The arrow at retention time 7.509 minutes marks the significantly enriched peak, later identified as valeric acid.

(a)

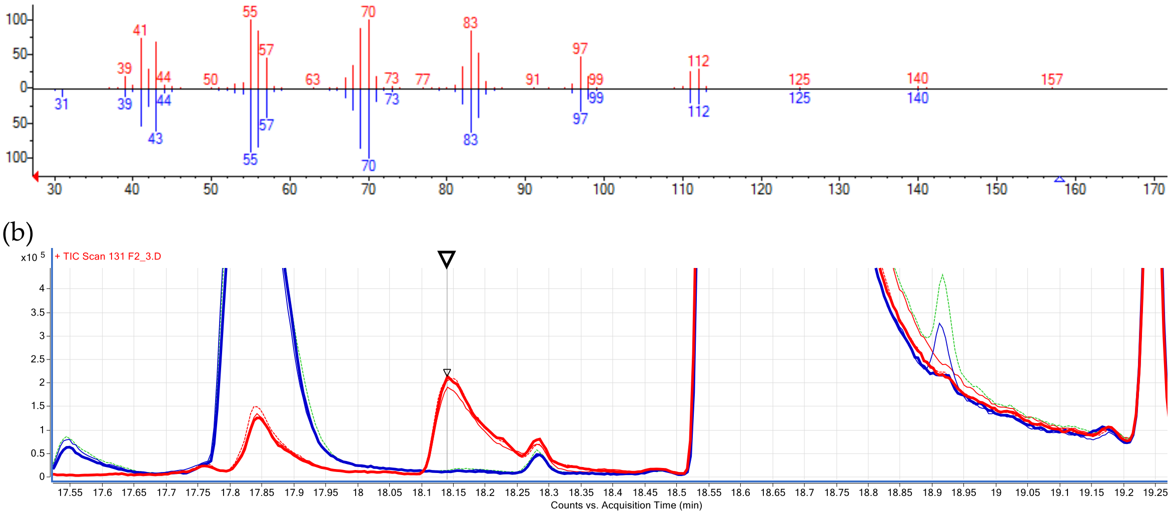

FIGURE S5. Detection of 1-decanol. (a) Comparison of the recorded mass spectrum (red) and the spectrum of the NIST reference (blue) for 1-decanol. (b) SPME GC-MS measurement displaying scans of the metagenomic clone (red) as compared to the empty vector strain *E. coli* EPI300 pCC2FOS (blue and green) with sample size n=3. The arrow at retention time 18.570 minutes marks the unique peak later identified as 1-decanol.

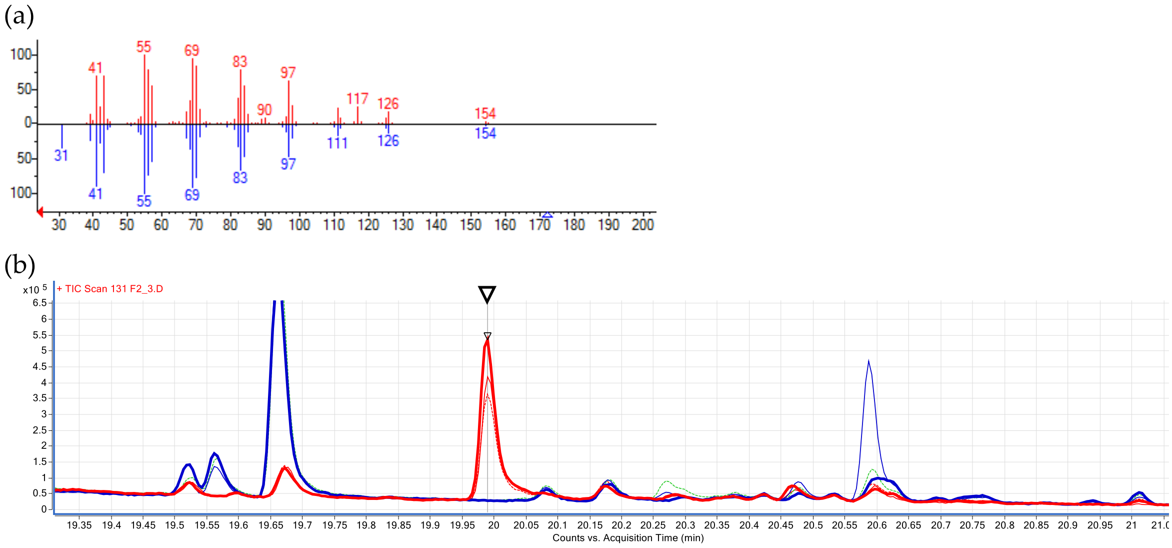

FIGURE S6. Detection of 1-undecanol. (a) Comparison of the recorded mass spectrum (red) and the spectrum of the NIST reference (blue) for 1-undecanol. (b) SPME GC-MS measurement displaying scans of the metagenomic clone (red) as compared to the empty vector strain *E. coli* EPI300 pCC2FOS (blue and green) with sample size n=3. The arrow at retention time 19.990 minutes marks the unique peak later identified as 1-undecanol.

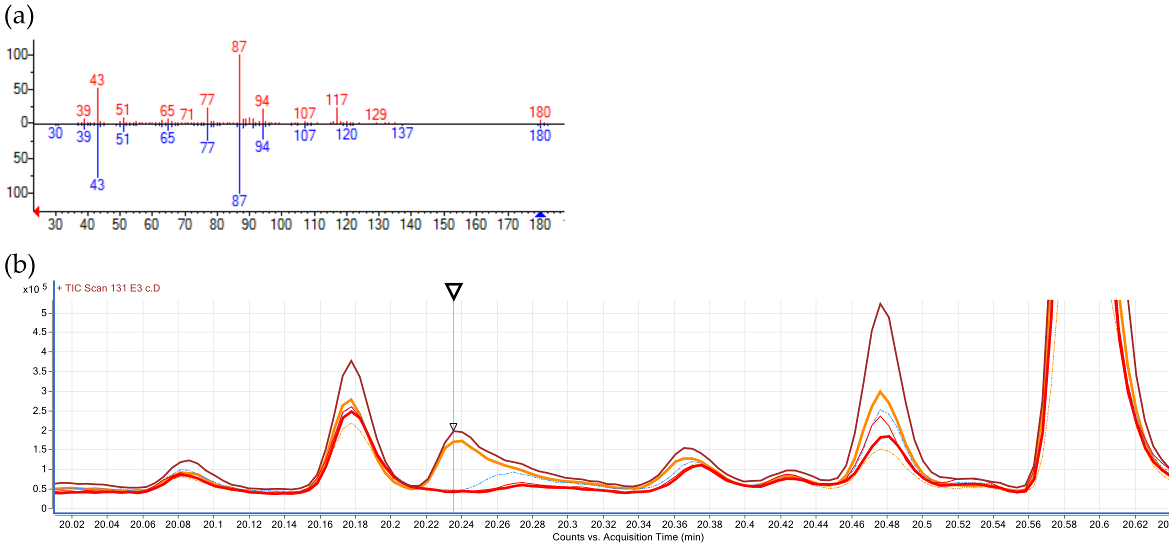

FIGURE S7. Detection of 2-phenoxyethyl acetate. (a) Comparison of the recorded mass spectrum (red) and the spectrum of the NIST reference (blue) for 2-phenoxyethyl acetate. (b) SPME GC-MS measurement displaying scans of the metagenomic clone (wine red to orange) as compared to the empty vector strain *E. coli* EPI300 pCC2FOS (blue and red) with sample size n=3. The arrow at retention time 20.235 minutes marks the unique peak later identified as 2-phenoxyethyl acetate.

(a)

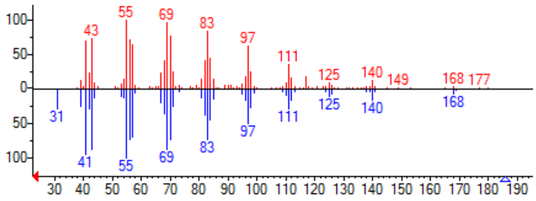

(b)

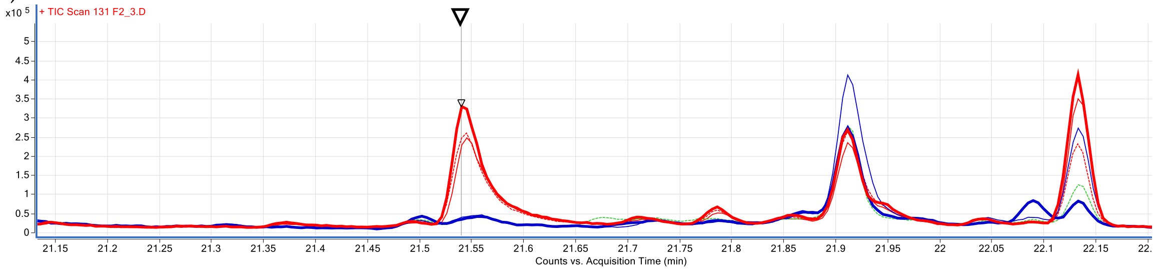

FIGURE S8. Detection of 1-dodecanol. (a) Comparison of the recorded mass spectrum (red) and the spectrum of the NIST reference (blue) for 1-dodecanol. (b) SPME GC-MS measurement displaying scans of the metagenomic clone (red) as compared to the empty vector strain *E. coli* EPI300 pCC2FOS (blue) with sample size n=3. The arrow at retention time 21.541 minutes marks the unique peak later identified as 1-dodecanol.

(a)

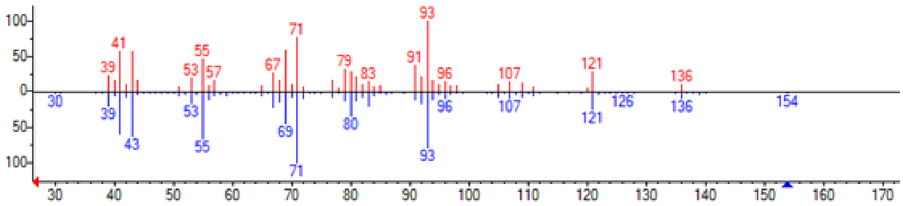

(b)

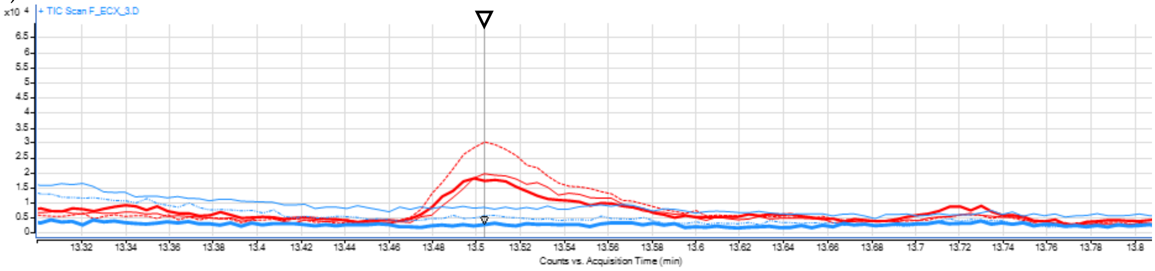

FIGURE S9. Detection of linalool. (a) Comparison of the recorded mass spectrum (red) and the spectrum of the NIST reference (blue) for linalool. (b) SPME GC-MS measurement displaying scans of the metagenomic clone (red) as compared to the empty vector strain *E. coli* EPI300 pCC2FOS (blue) with sample size n=3. The arrow at retention time 13.504 minutes marks the unique peak later identified as linalool.

(a)

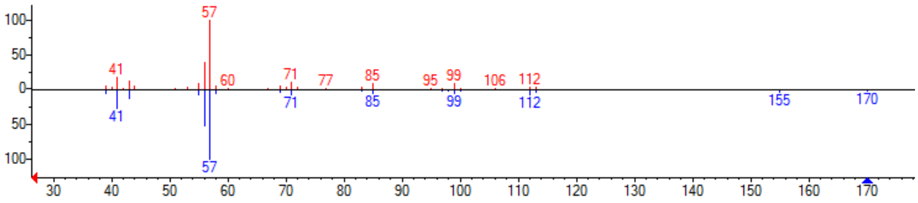

(b)

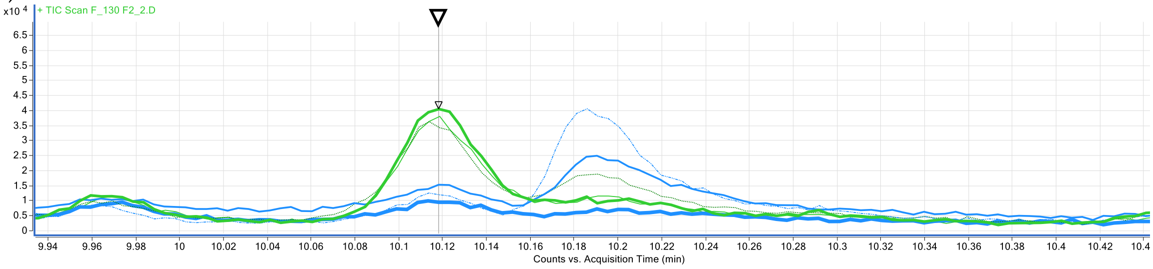

FIGURE S10. Detection of 2,2,4,6,6-pentamethylheptane. (a) Comparison of the recorded mass spectrum (red) and the spectrum of the NIST reference (blue) for 2,2,4,6,6-pentamethylheptane. (b) SPME GC-MS measurement displaying scans of the metagenomic clone (green) as compared to the empty vector strain *E. coli* EPI300 pCC2FOS (cyan) with sample size n=3. The arrow at retention time 10.119 minutes marks the unique peak later identified as 2,2,4,6,6-pentamethylheptane.

(a)

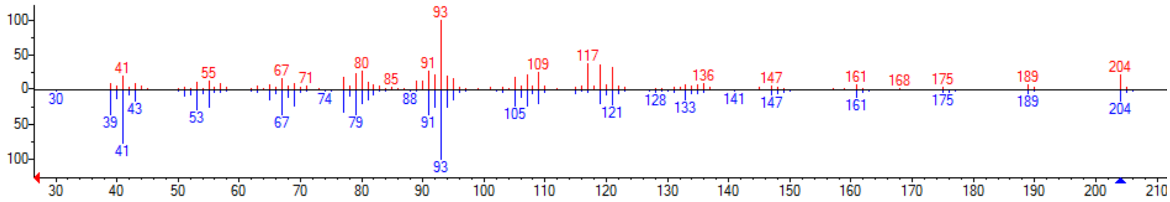

(b)

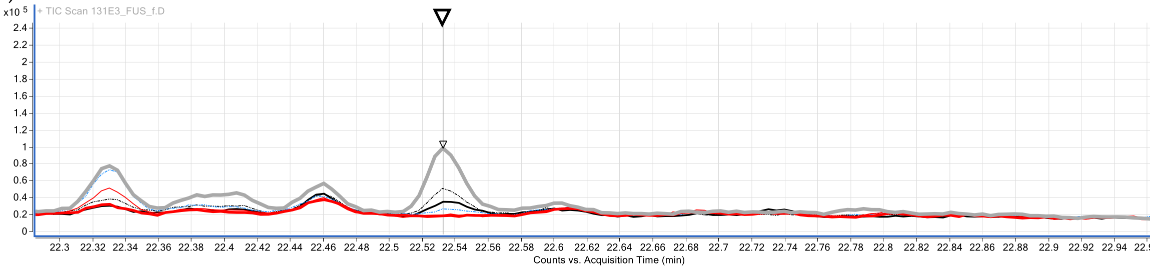

FIGURE S11. Detection of α-bisabolene. (a) Comparison of the recorded mass spectrum (red) and the spectrum of the NIST reference (blue) for α-bisabolene. (b) SPME GC-MS measurement displaying scans of the metagenomic clone (blue, grey and black) as compared to the empty vector strain *E. coli* EPI300 pCC2FOS (red) with sample size n=3. The arrow at retention time 22.533 minutes marks the unique peak later identified as α-bisabolene.

(a)

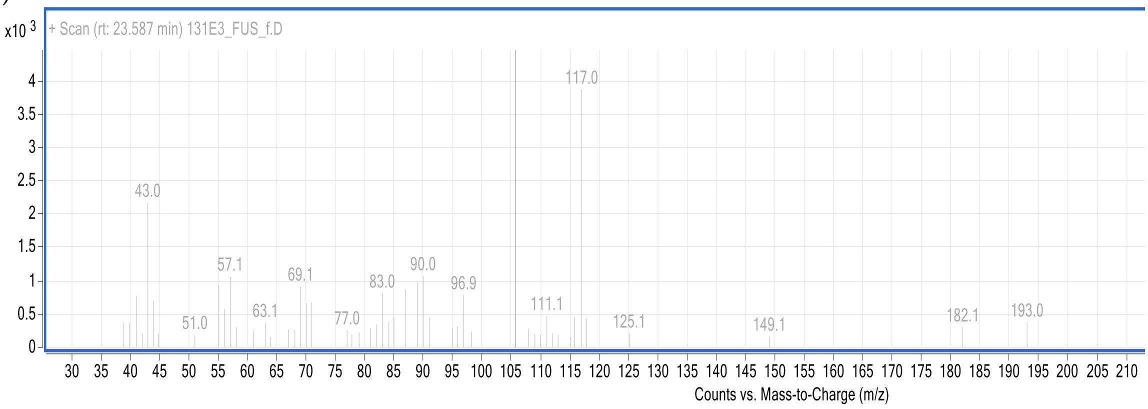

(b)

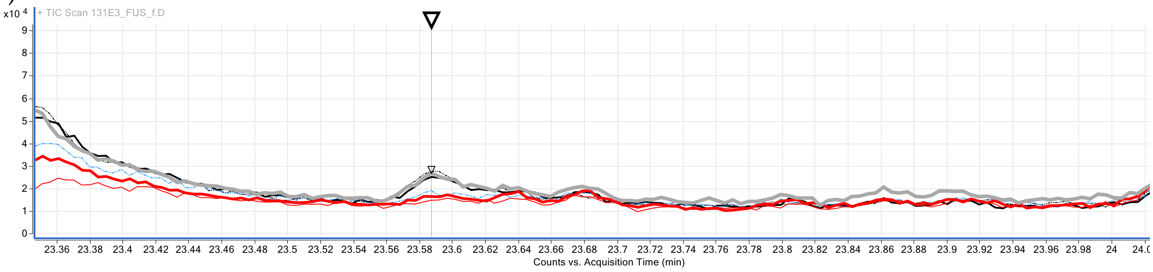

FIGURE S12. Detection of a not identified substance produced during interaction between *F. culmorum* and metagenomic library clone 131 E3. (a) The mass spectrum of the measured substance is depicted. (b) SPME GC-MS measurement displaying scans of the metagenomic clone (blue, grey and black) as compared to the empty vector strain *E. coli* EPI300 pCC2FOS (red) with sample size n=3. The arrow at retention time 23.587 minutes marks the unique peak later identified as an unknown substance.

TABLES

Table S1. Inhibition efficacy for different concentrations of the detected substances against *F. culmorum*.

Substance	Amount of substance (µmol)	Concentration (mM)	Inhibition Rate (%)	Standard deviation (%)
**1-Undecanol**	0.1	0.7	0	0
	1	6.7	0	0
	25	166.7	0	0
	50	333.3	0	0
	100	666.7	0	0
	250	1,666.7	0	0
	500	3,333.3	3.1	5.4
	716	4,773.3	48.2	5.6
**1-Decanol**	0.1	0.7	0	0
	1	6.7	1.8	2.5
	25	166.7	0.8	0.7
	50	333.3	0	0
	100	666.7	1.2	1.2
	250	1,666.7	1.6	1.8
	500	3,333.3	5.1	3.8
	770	5,133.3	51.8	4.6
**1-Dodecanol**	0.1	0.7	2.4	0
	1	6.7	0.4	0.7
	25	166.7	1.6	1.8
	50	333.3	2.0	3.4
	100	666.7	3.5	6.1
	250	1,666.7	10.2	3.6
	500	3,333.3	12.9	1.7
	657	4,380.0	14.3	9.3
**2-Phenoxyethyl acetate**	0.1	0.7	4.7	2.0
	1	6.7	0.4	0.7
	25	166.7	1.6	2.7
	50	333.3	0.4	0.7
	100	666.7	1.2	1.2
	250	1,666.7	2.4	2.0
	500	3,333.3	2.4	1.2
	933	6,220.0	25.0	7.1
**α-Bisabolol**	0.1	0.7	0	0
	1	6.7	0	0
	25	166.7	0	0
	50	333.3	0	0
	100	666.7	0.4	0.7
	250	1,666.7	0.6	0.8

	500	3,333.3	11.8	6.1
	503	3,353.3	11.5	2.3
**Linalool**	0.1	0.7	5.1	3.6
	1	6.7	17.6	5.4
	25	166.7	38.8	7.3
	50	333.3	69.0	6.9
	100	666.7	84.7	7.1
	250	1,666.7	100	0
	500	3,333.3	100	0
	812	5,413.3	100	0
**2,2,4,6,6-Pentamethylheptane**	0.1	0.7	0.4	0.7
	1	6.7	0.8	1.4
	25	166.7	0	0
	50	333.3	0	0
	100	666.7	0	0
	250	1,666.7	0	0
	500	3,333.3	0	0
	647	4,313.3	11.3	2.6
**Valeric acid**	0.1	0.7	10.2	7.7
	1	6.7	100	0
	25	166.7	100	0
	50	333.3	100	0
	100	666.7	100	0
	250	1,666.7	100	0
	500	3,333.3	100	0

References

[1] Obermeier, M.M.; Wicaksono, W.A.; Taffner, J.; Bergna, A.; Poehlein, A.; Cernava, T.; Lindstaedt, S.; Lovric, M.; Müller Bogotá, C.A.; Berg, G. Plant resistome profiling in evolutionary old bog vegetation provides new clues to understand emergence of multi-resistance. *ISME J* (under review).

[2] Bolger, A.M.; Lohse, M.; Usadel, B. Trimmomatic: a flexible trimmer for Illumina sequence data. *Bioinformatics* **2014**, *30*, 2114–20.

[3] Bankevich, A.; Nurk, S.; Antipov, D.; Gurevich, A.A.; Dvorkin, M.; Kulikov, A.s:; Lesin,, V.M.; Nikolenko, S.I.; Pham, S.; Prjibelski, A.D.; Pyshkin, A.V.; Sirotkin, A.V.; Vyahhi, N.; Tesler, G.; Alekseyev, M.A.; Pevzner, P.A. SPAdes: a new genome assembly algorithm and its applications to single-cell sequencing. *J Comput Biol* **2012**, *19*, 455–77.

[4] Seemann T. Prokka: rapid prokaryotic genome annotation. *Bioinformatics* **2014**, *30*, 2068–9.
